# Knee osteoarthritis induces atrophy and neuromuscular junction remodeling in the quadriceps and tibialis anterior muscles of rats

**DOI:** 10.1038/s41598-019-42546-7

**Published:** 2019-04-24

**Authors:** Jonathan Emanuel Cunha, Germanna Medeiros Barbosa, Paula Aiello Tomé de Souza Castro, Beatriz Leite Ferreira Luiz, Andreza Cristine Arcari Silva, Thiago Luiz Russo, Fernando Augusto Vasilceac, Thiago Mattar Cunha, Fernando Queiróz Cunha, Tania Fátima Salvini

**Affiliations:** 1 Physical Therapy Department, Federal University of São Carlos, São Carlos, SP Brazil; 2 Gerontology Department, Federal University of São Carlos, São Carlos, SP Brazil; 3 Pharmacology Department, University of São Paulo, Ribeirão Preto, SP Brazil

**Keywords:** Skeletal muscle, Osteoarthritis

## Abstract

Knee osteoarthritis (KOA) is associated with muscle weakness, but it is unclear which structures are involved in the muscle changes. This study assessed morphological alterations and the expression of genes and proteins linked to muscular atrophy and neuromuscular junctions (NMJs) in KOA, induced by anterior cruciate ligament transection (ACLT) in rats. Two groups of rats were assessed: control (without intervention) and KOA (ACLT surgery in the right knee). After 8 weeks, quadriceps, tibialis anterior (TA) and gastrocnemius muscles were analyzed (area of muscle fibers, NMJ, gene and protein expression). KOA group showed atrophy in quadriceps (15.7%) and TA (33%), with an increase in atrogin-1 and muscle RING-finger protein-1 (MuRF-1). KOA group showed quadriceps NMJ remodeling (reduction area and perimeter) and decrease in NMJ diameter in TA muscle. The expression of nicotinic acetylcholine receptor (nAChR) γ-nAChR increased and that of α-nAChR and muscle specific tyrosine kinase (MuSK) declined in the quadriceps, with a decrease in ε-nAChR in TA. MuRF-1 protein expression increased in quadriceps and TA, with no changes in neural cell adhesion molecule (NCAM). In conclusion, ACLT-induced KOA promotes NMJ remodeling and atrophy in quadriceps and TA muscles, associated with inflammatory signs and changes in muscle gene and protein expression.

## Introduction

Knee osteoarthritis (KOA) is a common disease that affects more than 250 million people worldwide^1^. It is a chronic, degenerative, inflammatory disorder^2^ that damages cartilage, narrows the intra-articular space and forms osteophytes^3^. Individuals with KOA exhibit pain, swelling, joint stiffness and loss of function^4^, with a decline in quality of life and functional capacity^5^. Anterior cruciate ligament transection (ACLT) in rats is considered a good model for studying KOA, since it promotes similar changes in human KOA^6^, such as deep erosion of joint cartilage and osteophyte formation^7^. Furthermore, studies have identified muscle fiber atrophy and increased atrogene expression, such as atrogina-1 and muscle RING-finger protein-1 (MuRF-1), in the quadriceps of rats, 15 days after ACLT^8^, while in rabbits, ACLT decreased knee extensor strength and reduced the electromyographic signal of quadriceps^9^.

Muscle weakness is common in KOA and considered a risk factor for disease development and progression^10^. The inflammatory characteristics of KOA lead to neural modifications such as pre-synaptic reflex inhibition, decreasing alpha motor neuron activation, causing atrophy and reducing muscle strength^11^. One of the most affected muscles in KOA are the quadriceps^12^, and their weakness accelerates cartilage destruction^10^ by promoting biomechanical changes in movement^13^ and raising joint overload^14,15^.

Changes in neuromuscular junctions (NMJs) may also be associated with muscle weakness in KOA, given that they are important for muscle function^16^. Chemical synapses occur between the motor neuron and muscle fibers, mediated by acetylcholine. Changes in NMJs or nicotinic acetylcholine receptors (nAChR), linked to muscle contraction, are known to compromise strength, resulting in muscle weakness and atrophy^17^. Despite the high incidence of KOA and associated muscle alterations, no studies to date have investigated possible NMJ changes in KOA that could contribute to elucidating the mechanisms associated with muscle weakness and atrophy. As such, the aim of this study was to assess the possible alterations in NMJs associated with muscle and functional changes in rats with ACLT-induced KOA.

## Results

### Morphometric analysis

**Figure 1:**
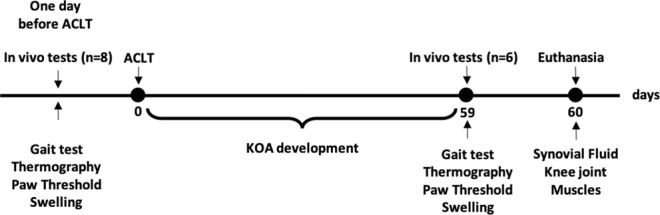
Experimental design. Representative figure of time and analyzes of the study. KOA = knee osteoarthritis, ACLT = anterior cruciate ligament transection.

60 days after ACLT (Fig. 1), the KOA group showed a decrease in the cross-sectional area (CSA) of the quadriceps (control 383.7 ± 22.04 μm^2^ vs KOA 319.4 ± 12.54 μm^2^; p = 0.029) and TA (control 425.2 ± 27.68 μm^2^ vs KOA 292.7 ± 27.16 μm^2^; p = 0.006; Table 1).

**Table 1:** Morphometric analysis of neuromuscular junctions and cross-sectional area muscle fiber of the quadriceps, tibialis anterior and gastrocnemius muscles.

Morphometric parameters	Control	KOA	p
**Quadriceps**			
NMJ Total area (µm²)	560.70 ± 80	346.72 ± 68	**0.001
NMJ Perimeter (µm)	315.10 ± 23	263.45 ± 21	**0.002
NMJ Maximum diameter (µm)	1194.96 ± 15	1123.66 ± 13	0.410
Muscle fiber CSA (µm²)	383.7 ± 22	319.4 ± 12	*0.029
**Tibialis anterior**			
NMJ Total area (µm²)	634.88 ± 86	613.04 ± 82	0.664
NMJ Perimeter (µm)	356.80 ± 86	340.62 ± 82	0.397
NMJ Maximum diameter (µm)	1371.53 ± 13	1143.46 ± 12	*0.014
Muscle fiber CSA (µm²)	425.2 ± 27	292.7 ± 27	**0.006
**Gastrocnemius**			
NMJ Total area (µm²)	624.08 ± 17	617.27 ± 60	0.929
NMJ Perimeter (µm)	130.81 ± 11	131.85 ± 11	0.877
NMJ Maximum diameter (µm)	1145.31 ± 80	1110.04 ± 15	0.626
Muscle fiber CSA (µm²)	425.2 ± 25	430.0 ± 52	0.935

Abbreviations: KOA = Knee osteoarthritis, NMJ = Neuromuscular junction, CSA = Cross-sectional area muscle fiber. Data are expressed as mean ± SEM. *p < 0.05 and **p < 0.001, KOA group compared to controls.

The NMJ of the quadriceps exhibited a decline in the area and perimeter (p = 0.001) in the KOA group compared to controls, while the TA only demonstrated a decline in the maximum NMJ diameter (p = 0.014) in the KOA group compared to controls. No changes in neuromuscular junctions were detected in the gastrocnemius muscle (Table 1).

**Figure 2:**
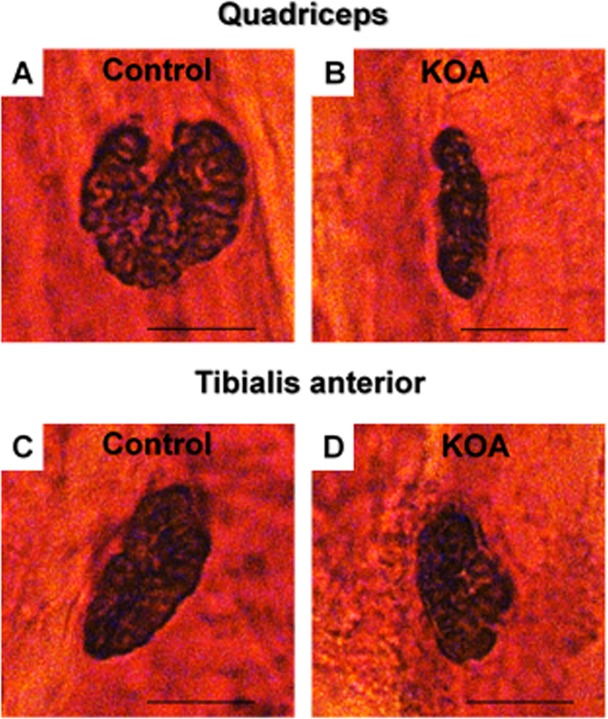
Morphology of neuromuscular junctions (Nonspecific Esterase Technique). Control group: quadriceps (**A**) and TA (**C**); KOA group: quadriceps (**B**) and TA (**D**). Abbreviations: KOA = Knee osteoarthritis group. Scale bar: 10 µm, 40X magnification.

In both the control and KOA groups, the NMJ showed fewer type pretzel branches, with normal morphological characteristics. This means a normal number of junctional folds that ensure the localization of nAChRs and the propagation of plaque potential to generate muscle contraction^18^. However, in the quadriceps muscle, there was a clear reduction in the spread of NMJs in the KOA group (Fig. 2B) compared to controls (Fig. 2A). The TA muscle showed a decrease in the maximum diameter (Fig. 2D) of the NMJ.

### Real-time (qPCR) PCR analysis of gene expression

**Figure 3:**
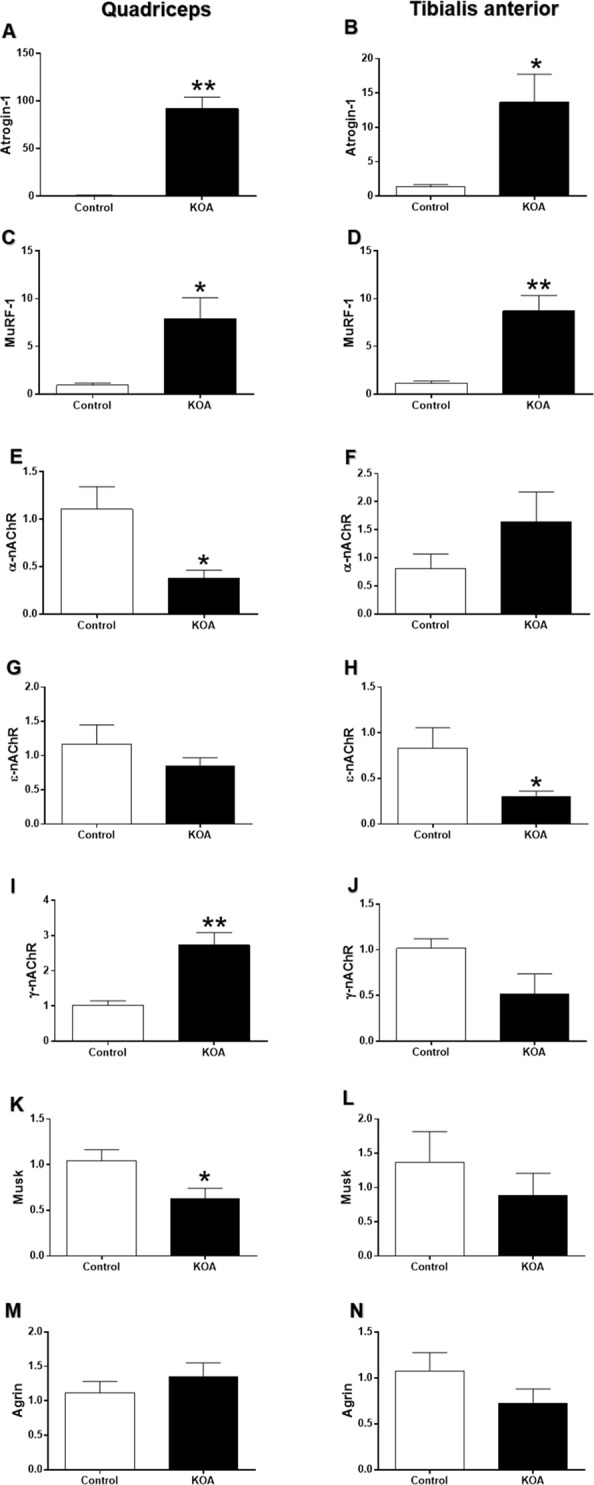
Gene expression analysis. mRNA expression in the quadriceps and TA muscles of control and KOA groups, respectively: Atrogin-1 (**A**,**B**); MuRF-1 (**C**,**D**); α-nAChR; (**E**,**F**); ε-nAChR; (**G**,**H**) γ-nAChR; (**I**,**J**); MuSK (**K**,**L**); Agrin (**M**,**N**). Abbreviations: KOA = Knee osteoarthritis. *p < 0.05; **p < 0.01, KOA group compared to controls.

An increase in atrogin-1 (p = 0.000) and MuRF-1 (p = 0.011) expression was observed in the quadriceps of the KOA group compared to controls. Moreover, α-nAChR (p = 0.016) and muscle-specific tyrosine kinase (MusK) (p = 0.031) expression declined and γ-nAChR (p = 0.001) expression in the KOA group rose in relation to controls (Fig. 3). For the TA, there was an increase in atrogin-1 (p = 0.013) and MuRF-1 (p = 0.001) expression, and a decline in ε-nAChR (p = 0.046) in the KOA group compared to controls (Fig. 3).

### Protein expressions by western blot analysis

**Figure 4:**
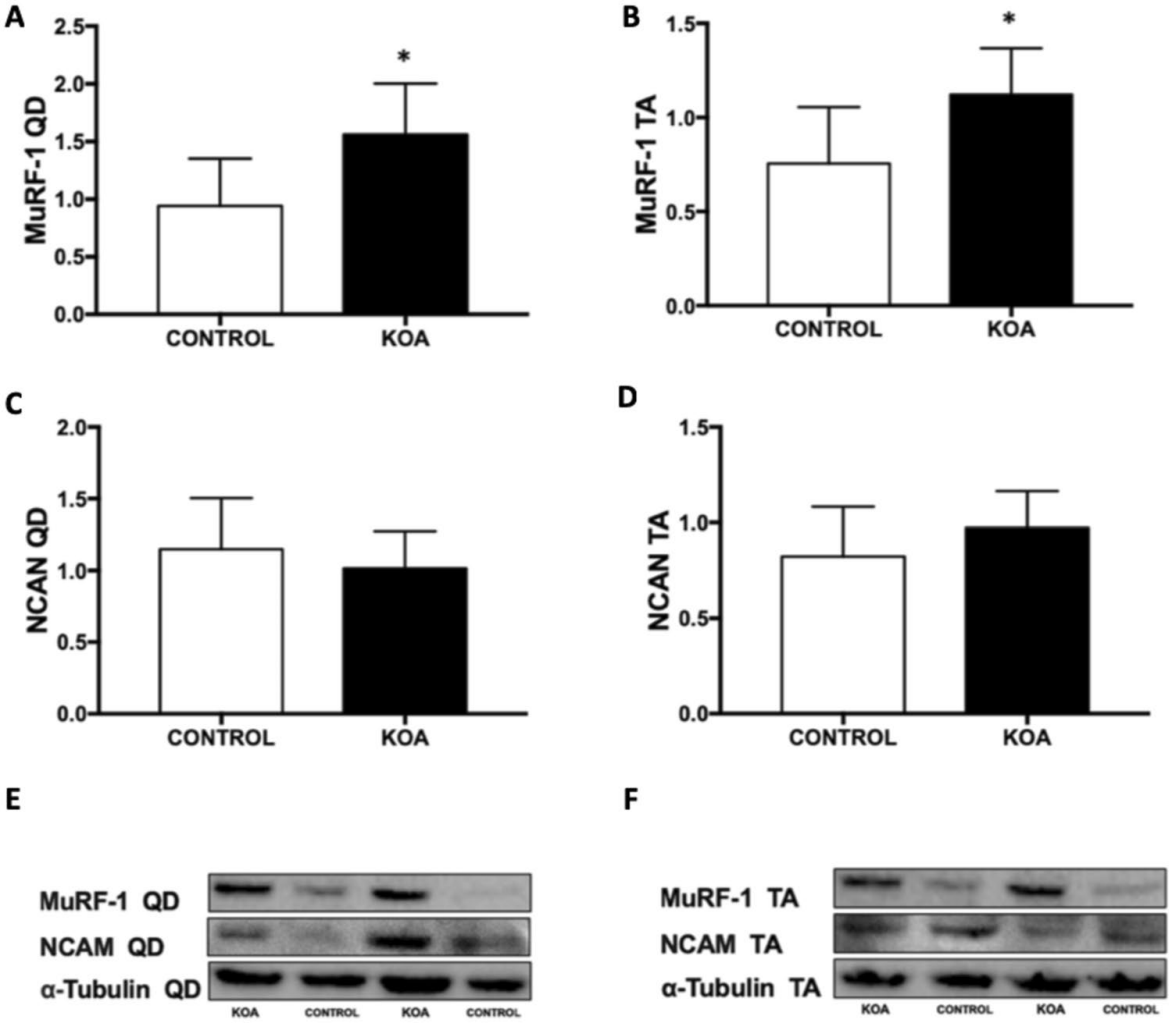
Protein Expression: Protein Expression of MuRF-1 and NCAM in the quadriceps (**A**,**C**,**E**) and TA (**B**,**D**,**F**) muscles. Abbreviations: KOA = Knee osteoarthritis group, QD = quadriceps muscle, TA = Tibialis anterior muscle, *p < 0.05, KOA group compared to controls.

An increase in MuRF-1 protein was found in KOA quadriceps (control 0.94.2 ± 0.16 vs KOA 1.56 ± 0.18; p = 0.03) and TA (control 0.75 ± 0.12 vs KOA 1.12 ± 0.10; p = 0.04) muscles compared to controls (Fig. 4). No difference was found in neural cell adhesion molecule (NCAM) protein in KOA quadriceps (control 1.15 ± 0.14 vs KOA 1.01 ± 0.10; p = 0.47) and TA (control 0.82 ± 0.10 vs KOA 0.97 ± 0.07 p = 0.28) muscles compared to controls (Fig. 4). The complete blots are described in Supplementary Figure 2.

### Gait test

**Figure 5:**
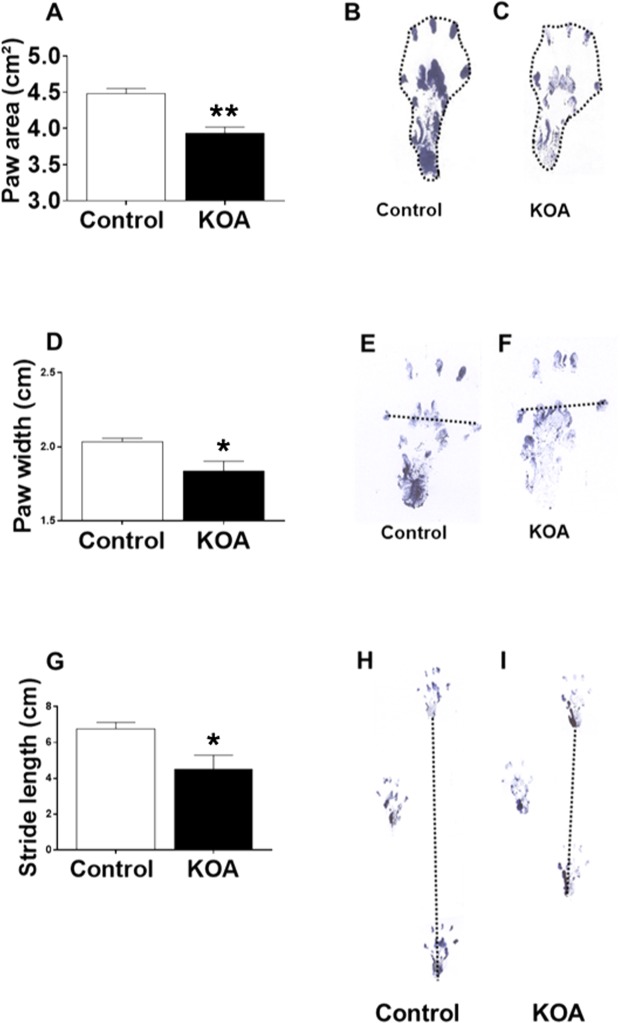
Gait test. Paw area (**A**): representation of the test in the control group (**B**) and KOA (**C**); Paw width (**D**): representation of the test in the control group (**E**) and KOA (**F**); Stride length (**G**): representation of the test in the control group (**H**) and KOA (**I**). Abbreviations: KOA = Knee osteoarthritis group. **p < 0.01 and *p < 0.05, KOA group compared to controls.

KOA reduced paw area (p = 0.000) and width (p = 0.023), with a decline in stride length (p = 0.016) compared to the control group (Fig. 5 and Supplementary Table 2).

### Inflammatory signs

**Figure 6:**
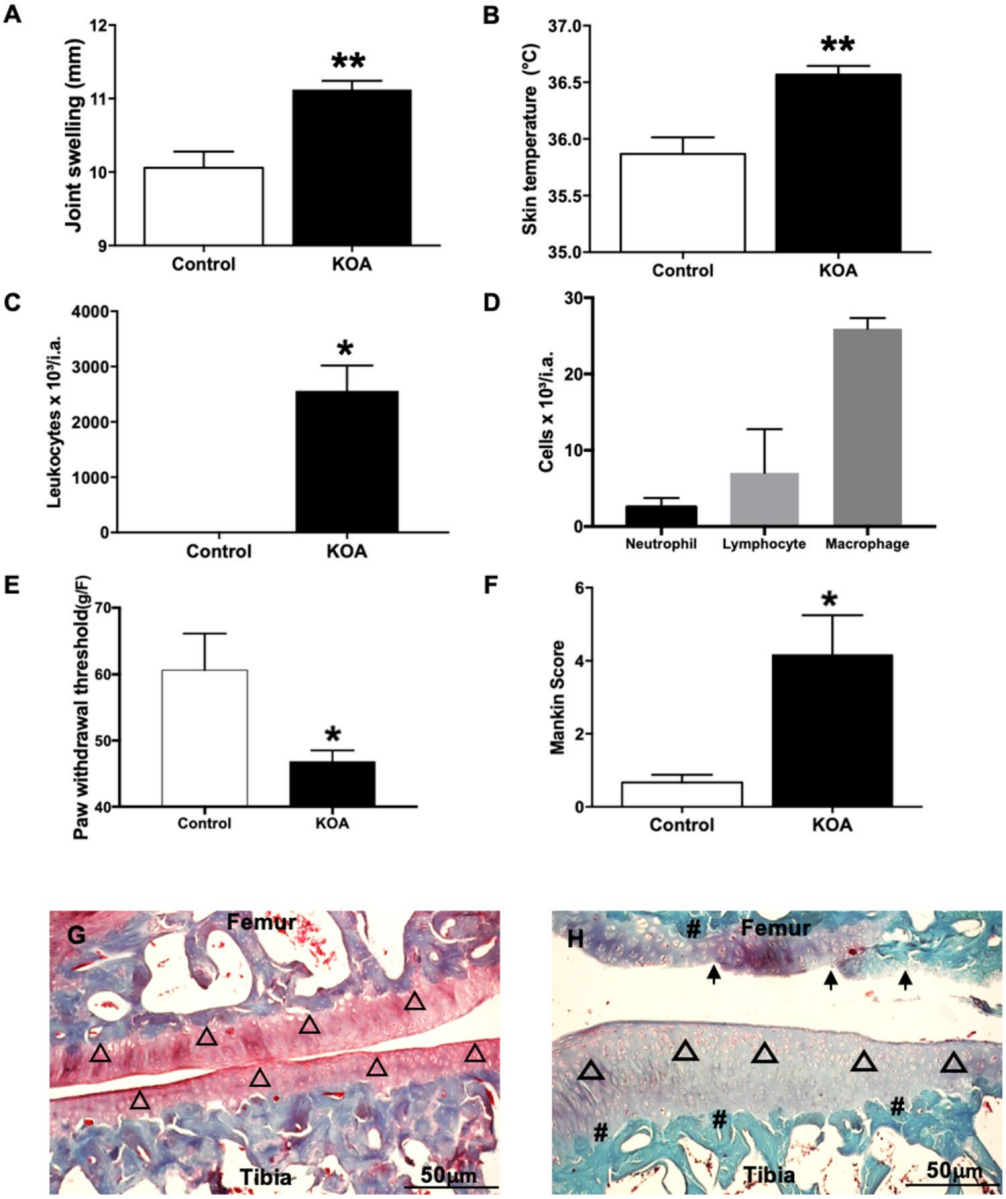
Inflammatory signs. Join swelling (**A**); Knee skin temperature (**B**); Leukocytes in synovial fluid (**C**); Differential cell count (**D**); Paw withdrawal threshold (**E**); Mankin score (**F**); Joint histology of the knee Safranin-O staining, representation of the control (**G**) and KOA group (**H**). KOA = Knee osteoarthritis; ▲ = joint surface with normal cells; ↑ = joint surface with irregularities; Δ = diffuse hypercellularity; # Reduction of Safranin O staining indicating severe reduction in proteoglycans. Scale bar = 50 µm, 20X magnification. **p < 0.01 and *p < 0.05, KOA group compared to control.

Joint swelling rose in the KOA group in relation to controls (11.1 ± 0.21 mm vs 10.0 ± 0.12 mm, respectively; p = 0.005; Fig. 6A). The KOA group showed an increase in the skin temperature of the knee compared to controls (36.56 ± 0.07 °C vs 35.82 ± 0.14 °C, respectively; p = 0.001), Fig. 6B. There was also a rise in leukocyte migration in the synovial fluid of the KOA group compared to controls (10.20 ± 1.86 × 10³/i.a. vs 0.0 ± 0 × 10³/i.a., respectively; p = 0.002; Fig. 6C). The differential count revealed a higher concentration of macrophages without synovial fluid compared to neutrophils and lymphocytes (Fig. 6D).

We found a decline in pain threshold in the Von Frey test in the KOA group compared to controls (46.82 ± 0.69 g/F vs 60.63 ± 2.25 g/F, respectively; p = 0.0002), Fig. 6E. The KOA group showed a higher Mankin histological score than that of controls (4.16 ± 1.07 vs 0.66 ± 0.21, respectively; p = 0.028; Fig. 6F and Supplementary Table 3). Figure 6G,H show a difference in proteoglycan content, evidenced by dye intensity (red), in addition to discontinuity on the joint surface in the KOA group.

## Discussion

This study provided a novel contribution, showing changes in NMJs associated with quadriceps and TA muscle atrophy in rats with KOA. The most significant NMJ changes (decreased area and perimeter) were observed in the quadriceps, which exhibited 15.7% atrophy in muscle fibers. Although the TA demonstrated greater muscle atrophy (33%), alterations in the NMJs were smaller than in the quadriceps and restricted to an increase in diameter. Increased MuRF-1 expression^19^ and muscle autophagy^20^ have been suggested as possible mechanisms associated with NMJ alterations. Our study also found an increase in MuRF-1 and atrogin-1 expression could be associated with NMJ changes. It is known that MuRF-1 and atrogin-1 are the main signalers of muscle atrophy in different experimental models^21,22^. We identified an increase in the protein expression of MuRF-1 in the quadriceps and TA muscles, in line with increased genetic expression, indicating ACLT-induced atrophy in the KOA model. In addition to being involved in muscle atrophy, recent studies suggest that MuRF-1 is an important mediator in regulating the amount of nAChRs in the NMJ^19,23^, and may also be linked to NCAM, a regulator of synaptic stability related to neurotransmission^24,25^. However, the KOA group showed no changes in the protein expression of NCAM, which may be more sensitive only in cases of muscle denervation^26,27^. However, the increase in MURF-1 protein expression observed in this model may be related to both muscle atrophy and NMJ changes.

Most studies with KOA focus on observing changes in quadriceps, since they are directly related to the knee joint and exhibit muscle atrophy^28–30^, reduced strength^14,31^ and functional decline^15,32^. In addition to muscle atrophy, the quadriceps showed greater NMJ alterations. With respect to mRNA expression in the quadriceps, we observed an increase in the gamma subunit of the acetylcholine receptor (γ-nAChR), which is normally expressed in the embryonic phase^33^, but also in muscle diseases such as denervation^34^. There was also a decline in alpha subunit (α-nAChR) expression, responsible for acetylcholine flow into the postsynaptic membrane^35^, as well as MuSK, a gene that initiates post-synaptic differentiation and plays an important clustering role and maintains nAChR^36^. The results of the present study show intense remodeling in the NMJs of quadriceps associated with KOA. Muscle fiber atrophy and arthrogenic muscle inhibition, which decreases quadriceps activation by afferent inhibition of the α motor neuron^11,37^, may be important mechanisms associated with the NMJ changes identified in this muscle. Another possible mechanism may be nerve terminal withdrawal, modulating the release of acetylcholine and leading to adaptation of the NMJs^38,39^. However, other studies are needed to assess the mechanisms involved in NMJ remodeling observed in the present study.

The TA muscle is little researched in KOA. We found only one study that assessed the TA of mice 4 and 8 weeks after KOA induction, exhibiting a decline in strength, 40% decrease in relaxation rate and less ATP expression, but with no alterations in the cross-sectional area of muscle fibers^40^. On the other hand, some studies on the TA of individuals with KOA assess function^41,42^ and report a decline in eccentric strength in ankle dorsiflexors^43^. However, there is no clear evidence about the TA atrophy mechanisms in individuals with KOA. We believe that the greater atrophy observed in our study may be related to disuse, as described for quadriceps^31^, due to muscle compensation^13^, or because TA is a monoarticular muscle that may be more affected by atrophy^44^. Given the significant atrophy and NMJ alterations observed in the TA muscle, we believe that this muscle deserves to be more thoroughly studied in the KOA. Future studies on TA adaptations in the KOA may provide new scientific evidence to clinical practice and rehabilitation of this muscle.

The absence of neuromuscular changes in the gastrocnemius muscle observed here indicates that the KOA affects primarily the knee extensor muscles such as the quadriceps^9,15^ and those that contribute to decelerate movement during functional activities such as the TA muscle^45,46^. Our results corroborate this hypothesis.

Our findings showed impaired gait pattern in the KOA group, as observed in similar studies^47,48^. Recent studies have associated gait deficits with a decrease in the CSA of the gastrocnemius muscle of rats^49^ and long extensor of mouse fingers^50^. In addition, changes in gait were also associated with increased KOA pain in mice^51^. Changes in the gait pattern of animals are similar to the functional changes observed in individuals with KOA^52^. Our results show a correlation between altered gait patterns and the neuromuscular changes identified by atrophy and fewer NMJs in the quadriceps and TA muscles of rats with KOA.

The paw withdrawal threshold declined in the KOA group. This result corroborates a recent study that found reduced pain threshold associated with muscle weakness after ACLT^53^. Another recent study showed the presence of persistent mechanical hyperalgesia in a KOA model^54^. Our results strengthen the evidence that pain is related to the inflammatory and neuromuscular changes observed in the KOA.

We found an increase in leukocytes (73% macrophages) in the synovial fluid of the KOA group. Our results corroborate earlier studies indicating that macrophages are the main component of synovial fluid cells in KOA, contributing to the destruction of cartilage and osteophyte growth^55,56^. Recent studies have also classified the KOA into subgroups related to macrophages^57^. In addition to increased leukocytes in the synovial fluid, we observed increases in other signs, thereby strengthening the role of inflammation in the pathogenesis of KOA, such as greater knee surface temperature and swelling.

## Materials and Methods

All experiments and procedures were performed in accordance with the *Guide for the Care and Use of Laboratory Animals* published by the U.S. National Institute of Health and approved by the Ethics Committee on the Use of Animals of Sao Carlos Federal University (Protocol number 9197020816). Trained professionals blinded to the identity of the experimental groups conducted all procedures.

### Experimental design and period

Rats Wistar (two months old, average weight of 214.41 ± 20 g) were randomly distributed into two groups (n = 6 per group): a) control (without surgery) and b) KOA (submitted to ACLT). Sample size was determined based on data from a pilot study, with muscle fiber cross-sectional area and NMJ area for quadriceps and TA muscles as the primary outcomes. Considering an alpha value of 0.05 and power of 95% (Software GPower 3.1), the sample size was calculated to be 5 animals per group. In the event of a possible loss during the experimental procedures, 8 animals were allocated to each group. Two animals from each group died during the experiments and the causes of death are described in Supplementary Table 1.

The rats were housed in cages (two animals per cage) in pathogen-free conditions at 24 °C ± 1 °C under a reverse light cycle (12 h light /12 h dark), with unrestricted access to standard rat chow and water. All animals underwent *in-vivo* tests one day before and 59 days after the ACLT and were euthanized the following day. The experimental design and study period are described in Fig. 1.

### ACLT-induced KOA

An adapted ACLT-induced KOA model was used. The rats were anesthetized with an intraperitoneal injection (12 mg/kg xylazine and 95 mg/kg ketamine). The right knee was shaved and prepared using an iodine solution. An incision on the medial side of the patellar tendon provided access to the joint space, after which the patella was dislocated laterally with the leg in extension. Afterwards, the joint capsule injury and ACLT were performed with ophthalmic scissors. Next, the anterior drawer test (free displacement of the tibia in relation to the femur) was conducted to confirm the ACLT^8,58^. The animals were returned to their cages and paracetamol (13.5 mg/100 mL) was added to the drinking water for the first 48 hours after surgery as postoperative analgesia. We used only the control group without surgical intervention because previous reports found joint wear similar to KOA in the sham groups (knee surgery without ACLT)^8,59^.

### Muscle sample collection

The quadriceps, tibialis anterior (TA) and gastrocnemius muscles were isolated, removed and weighed. Each muscle was divided into three parts. The proximal fragment was immersed in Isopentane (pre-cooled in liquid nitrogen), stored at −80 °C, and used to measure the cross-sectional area (CSA) of the muscle fibers. The middle fragment was used for the nonspecific esterase technique. The distal fragment was rapidly frozen in liquid nitrogen, stored at −80 °C, and used to measure mRNA and protein levels

### Muscle Fiber CSA

Histologic serial cross-sections were obtained from the quadriceps, TA and gastrocnemius muscles in a cryostat microtome (Leica, CM1860, *Germany*). A histologic cross-section (10 µm) stained with toluidine blue was selected to measure the CSA under a light microscope (*Axiovision 3*.*0*.*6 SP4 - Carl Zeiss*, *Germany*) using morphometric analysis (Image J software, version 1.43u, National Institutes of Health, USA). The CSA of each muscle was obtained by measuring 100 fibers located in the central region of the section. The percentage atrophy index was calculated by the difference in the proportion between the cross-sectional area of muscle fibers from the control group and the cross-sectional area of the KOA muscle fibers. The atrophy index was calculated by the weight of each muscle (g) and normalized using body mass (BM; g) to obtain the MM/BM ratio of the control and KOA groups^8^.

### NMJ analysis - nonspecific esterase technique

The surface portions of the quadriceps, TA and gastrocnemius muscles were trimmed to the motor end-plate portion (containing the motor point), which was cut lengthwise into three or four slices. The resulting material was subjected to the nonspecific esterase technique^60^ to characterize the NMJ.

### Morphometric analysis

Maximum diameter, total area and perimeter were measured on 30 junctions with a light microscope (Axiovision 3.0.6 SP4 - Carl Zeiss, Germany). Two experienced blinded observers analyzed the images with Image J software (version 1.43u, National Institutes of Health, USA)^61,62^.

### Analysis of mRNA Expression by Real-Time PCR

Total RNA was extracted from quadriceps and TA muscle samples in the control and KOA groups for mRNA analysis using TRIzol Reagent (Life Technologies, USA), according to the manufacturer’s recommendations. The RNA was quantified using a Bioresearch BioPhotometer spectrophometer (Eppendorf® Hamburg, Germany), which also determined RNA purity by measuring absorbance at 260 nm (RNA quantity) and 280 nm (protein quantity). Only samples with 260/280 ratios > 1.8 were used. RNA integrity was evaluated by ethidium bromide staining (Invitrogen) based on 28s and 18s ribosomal RNAs. Extracted RNA was treated with DNase I, Amplification Grade (Sigma Aldrich, AMPD1) to eliminate any possible contamination with genomic DNA from the samples. mRNA reverse transcription was performed using the iScript^TM^ cDNA Synthesis Kit (Bio-Rad, CA), following the manufacturer’s guidelines. The expression levels of mRNAs were assessed by quantitative real-time PCR (qPCR) using the *CFX 96* Touch^TM^ Real Time PCR Detection System, version 3.0 (Bio-Rad, CA). The cDNA samples corresponding to the mRNA of the genes analyzed were amplified by SsoFast™ EvaGreen^®^ Supermix (Bio-Rad, CA) and primers were designed using Primer Express® 3.0.11 software (Applied Biosystems, CA, USA) from sequences published in GenBank (www.pubmed.com) and synthesized by Life Technologies (USA) (Supplementary Table 5). The expression levels were normalized by glyceraldehyde 3-phosphate dehydrogenase (GAPDH), peptidylprolyl isomerase A (PPLA), peptidylprolyl isomerase B (PPLB), beta cytoskeletal actin (ACTB) and hypoxanthine–guanine phosphoribosyltransferase (HPRT), whose expression was constant among all samples. Relative quantification of gene expression was performed using the comparative 2^∆∆^C(T) method^63^.

### Protein expressions by western blot analysis

Protein samples (containing 80 μg of protein) were collected from the quadriceps and TA muscles. These were separated on SDS/PAGE gels (12% wt/vol) and transferred to a nitrocellulose membrane. The membranes were incubated with antibodies against MuRF-1 (IgG, polyclonal, 1:500, 40Kda, Gene-Tex, USA) NCAM (IgG, polyclonal, 1:1000, 200Kda, Merck, USA) and normalized using α-Tubulin (IgG, monoclonal, 1:1000, 50Kda, Sigma, USA). The blots were visualized in an ECL solution (Amersham Pharmacia Biotech, Little Chalfont, UK) and exposed in a ChemiDoc MP Imaging System (Bio-Rad Laboratories, Hercules, California, USA)^64,65^.

### Gait test

The hind paws of the rats were brushed with ink. Next, the animals were allowed to run on a 60 cm-long, 7 cm-wide track covered with white paper. A dark chamber was placed at the end of the track to entice the rats. Upon completion of the test, the paper was scanned at 300 dpi^47^. The measurement around the paw was defined as paw area (cm²), the distance between the first and fifth toes as paw width (cm), the distance of the same hind paw between two steps as stride length (cm), the horizontal distance between the left and right paw as the base (cm), the distance between the third toe and the heel as paw length (cm), and the paw angle as the angle through the hind legs (°). The measures of footsteps were quantified by ImageJ software (version 1.43u, National Institutes of Health, USA).

### Joint swelling (edema)

Three measures of knee joint thickness were taken under anesthesia (0_2_: 2.0 L/m, 2% isoflurane), using an electronic digital caliper (Mitutoyo Absolute Digimatic 150 mm, Japan). The results were expressed in mm^65^.

### Knee skin temperature

The animals were acclimated in a dark room (15 min; 24 °C ± 1). Thermography was used to quantify the skin temperature of the knee in both KOA and control groups using an infrared thermal camera (FLIR Systems® T420, USA), placed on a tripod 50 cm from the animal’s knee. The images were analyzed in Flir Tools software, and the results expressed in (°C).

### *Ex-vivo* leukocyte migration

Leukocyte migration was determined by synovial fluid, as previously described^66,67^. The joint cavities were washed twice with 5 μL phosphate-buffered saline (PBS) solution containing 1 mM ethylenediaminetetraacetic acid (EDTA) and then diluted to a final volume of 50 μL with PBS/EDTA to evaluate leukocyte migration at the indicated time. The total number of leukocytes was counted in a Neubauer chamber diluted in Turk’s solution. The results were expressed as the number of leukocytes per joint cavity. Differential cell counts were determined in cytocentrifuge Rosenfeld-stained slices (Cytospin 4; Shandon, Pittsburgh, PA, USA). Differential cell counts were performed with a light microscope, and the results were expressed as the number (mean ± SEM) of leukocytes per joint cavity. For this analysis we used only 4 animals from the KOA group^66^.

### Paw withdrawal threshold

The articular hypernociception of the femur-tibial joint was evaluated using an Electronic Von Frey meter (*Insight*® EFF-301). The animals were acclimated (30 min) in acrylic cages with a wire grid floor (12 × 10 × 17 cm high). A trained investigator (blind to group allocation) applied perpendicular pressure to the plantar surface of the hind paw. When the paw was withdrawn, the intensity of the force applied was automatically recorded. The mechanical threshold results are expressed in grams (g/F)^53,66^.

### Histological assessment of the knee joint

A standard histological protocol was used. Briefly, the knees were fixed (4% formaldehyde for 2 days) and decalcified (10% EDTA). The samples were embedded in paraffin blocks and histological sections were obtained (10 μm) using a microtome (Leica RM-2245, Germany). Samples were stained with hematoxylin and eosin (HE-Merck, Darmstadt, Germany) and 0.1% Safranin-O (Merck, Darmstadt, Germany)^68,69^, and examined under a light microscope (×100; Axiovision 3.0.6 SP4 - Carl Zeiss, Germany). Two experienced blinded observers evaluated cartilage damage using the Modified Mankin Score^70^, Supplementary Table 4.

### Statistical analysis

Continuous variables were presented as mean ± standard error of the mean (SEM). Since all variables were normally distributed (p > 0.05), according to the Shapiro-Wilk test, an independent t-test was performed. Statistical analyses were performed with SPSS, version 23.0 (SPSS Inc., Chicago, USA). The figures were plotted in GraphPad Prism software, version 5.0. A p-value < 0.05 was considered statistically significant.

## Conclusion

ACLT-induced KOA in rats promotes NMJ remodeling and atrophy in quadriceps and TA muscles, associated with inflammatory signs and alterations in gait and muscle gene and protein expression.

## Supplementary information

Supplementary table 1, Supplementary table 2, Supplementary table 3, Supplementary table 4, Supplementary table 5; Supplementary Figure 1; Supplementary Figure 2

## Data Availability

The datasets generated and/or analyses conducted during the study are available from the corresponding author.

## References

[1] Vos, T. *et al*. Years lived with disability (YLDs) for 1160 sequelae of 289 diseases and injuries 1990–2010: A systematic analysis for the Global Burden of Disease Study 2010. *Lancet* **380**, 2163–2196 (2012). 10.1016/S0140-6736(12)61729-2 PMC6350784 23245607

[2] Glyn-Jones S (2015). Osteoarthritis. Lancet.

[3] Loeser RF, Goldring SR, Scanzello CR, Goldring MB (2012). Osteoarthritis: A disease of the joint as an organ. Arthritis Rheum..

[4] Sharma L (2016). Osteoarthritis year in review 2015: clinical. Osteoarthr. Cartil..

[5] Neogi T (2013). The Epidermiology and Impact of Pain in Osteoarthritis. Osteoarthr. Res. Soc..

[6] Fang H, Beier F (2014). Mouse models of osteoarthritis: modelling risk factors and assessing outcomes. Nat. Rev. Rheumatol..

[7] Loeser RF (2013). Disease Progression and Phasic Changes in Gene Expression in a Mouse Model of Osteoarthritis. PLoS One.

[8] Delfino GB (2013). Quadriceps muscle atrophy after anterior cruciate ligament transection involves increased mRNA levels of atrogin-1, muscle ring finger 1, and myostatin. Am. J. Phys. Med. Rehabil..

[9] Herzog W, Longino D, Clark A (2003). The role of muscles in joint adaptation and degeneration. Langenbeck’s Arch. Surg..

[10] Roos EM, Herzog W, Block Ja, Bennell KL (2011). Muscle weakness, afferent sensory dysfunction and exercise in knee osteoarthritis. Nat. Rev. Rheumatol..

[11] Hurley MV, Newham DJ (1993). The influence of arthrogenous muscle inhibition on quadriceps rehabilitation of patients with early, unilateral osteoarthritic knees. Rheumatology.

[12] Terracciano C (2013). Differential features of muscle fiber atrophy in osteoporosis and osteoarthritis. Osteoporos. Int..

[13] Igawa T, Katsuhira J (2014). Biomechanical Analysis of Stair Descent in Patients with Knee Osteoarthritis. J. Phys. Ther. Sci..

[14] Amin S (2009). Quadriceps strength and the risk of cartilage loss and symptom progression in knee osteoarthritis. Arthritis Rheum..

[15] Rice DA, McNair PJ, Lewis GN (2011). Mechanisms of quadriceps muscle weakness in knee joint osteoarthritis: The effects of prolonged vibration on torque and muscle activation in osteoarthritic and healthy control subjects. Arthritis Res. Ther..

[16] Nudell BM, Grinnell aD (1983). Regulation of synaptic position, size, and strength in anuran skeletal muscle. J. Neurosci..

[17] Hughes BW, Kusner LL, Kaminski HJ (2006). Molecular architecture of the neuromuscular junction. Muscle Nerve.

[18] Engel, A. G. & Franzini-Armstrong, C. *Myology* (2004).

[19] Rudolf R (2013). Regulation of nicotinic acetylcholine receptor turnover by MuRF1 connects muscle activity to endo/lysosomal and atrophy pathways. Age (Omaha)..

[20] Carnio S (2014). Autophagy Impairment in Muscle Induces Neuromuscular Junction Degeneration and Precocious Aging. Cell Rep..

[21] Bonaldo P, Sandri M (2013). Cellular and molecular mechanisms of muscle atrophy. Dis. Model. Mech..

[22] Gumucio JP, Mendias CL (2013). Atrogin-1, MuRF-1, and sarcopenia. Endocrine.

[23] Rodrigues ACZ (2018). The Sympathetic Nervous System Regulates Skeletal Muscle Motor Innervation and Acetylcholine Receptor Stability. Acta Physiol.

[24] Enriquez-barreto L, Palazzetti C, Brennaman LH, Maness PF (2012). Neural cell adhesion molecule, NCAM, regulates thalamocortical axon pathfinding and the organization of the cortical somatosensory representation in mouse. Frontiers in Molecular Neuroscience.

[25] Chattopadhyaya B, Baho E, Huang ZJ, Schachner M, Cristo G (2013). Di. Neural Cell Adhesion Molecule-Mediated Fyn Activation Promotes GABAergic Synapse Maturation in Postnatal Mouse Cortex. The Journal of Neuroscience.

[26] Pinheiro-Dardis CM, Erbereli BT, Gigo-Benato D, Castro PATS, Russo TL (2017). Electrical stimulation delays reinnervation in denervated rat muscle. Muscle Nerve.

[27] Hendrickse P, Galinska M, Hodson-tole E, Degens H (2018). An evaluation of common markers of muscle denervation in denervated young-adult and old rat gastrocnemius muscle. Exp. Gerontol..

[28] Herzog W, Longino D (2007). The role of muscles in joint degeneration and osteoarthritis. J. Biomech..

[29] Goodman SM (2017). American College of Rheumatology/American Association of Hip and Knee Surgeons Guideline for the Perioperative Management of Antirheumatic Medication in Patients With Rheumatic Diseases Undergoing Elective Total Hip or Total Knee Arthroplasty. J. Arthroplasty.

[30] Neill TWO, Mccabe PS, Mcbeth J (2018). Update on the epidemiology, risk factors and disease outcomes of osteoarthritis. Best Pract. Res. Clin. Rheumatol.

[31] Felson DT (2000). Osteoarthritis: new insights. Part 1: the disease and its risk factors. Ann. Intern. Med..

[32] Serrao PRMS, Gramani-Say K, Lessi GC, Mattiello SM (2012). Knee extensor torque of men with early degrees of osteoarthritis is associated with pain, stiffness and function. Rev. Bras. Fisioter..

[33] Missias AC, Chu GC, Klocke BJ, Sanes JR, Merlie JP (1996). Maturation of the Acetylcholine Receptor in Skeletal Muscle: Regulation of the AChR γ-to-ϵ Switch. Dev. Biol..

[34] Hesselmans LFGM, Jennekens FGI, Van Den Oord CJM, Veldman H, Vincent A (1993). Development of innervation of skeletal muscle fibers in man: Relation to acetylcholine receptors. Anat. Rec..

[35] Dani JA (2015). Neuronal Nicotinic Acetylcholine Receptor Structure and Function and Response to Nicotine. Int Rev NeuroBiol.

[36] Strochlic L, Cartaud A, Cartaud J (2005). The synaptic muscle-specific kinase (MuSK) complex: New partners, new functions. BioEssays.

[37] Palmieri-Smith RM, Thomas AC (2009). A neuromuscular mechanism of posttraumatic osteoarthritis associated with ACL injury. Exerc. Sport Sci. Rev..

[38] Khan MAS (2014). Nonsurgically induced disuse muscle atrophy and neuromuscular dysfunction upregulates alpha7 acetylcholine receptors. Can. J. Physiol. Pharmacol..

[39] Melroy-Greif WE, Stitzel JA, Ehringer MA (2016). Nicotinic acetylcholine receptors: upregulation, age-related effects and associations with drug use. Genes, Brain Behav..

[40] van der Poel C, Levinger P, Tonkin BA, Levinger I, Walsh NC (2016). Impaired muscle function in a mouse surgical model of post-traumatic osteoarthritis. Osteoarthr. Cartil..

[41] Resende RA, Kirkwood RN, Deluzio KJ, Morton AM, Fonseca ST (2016). Mild leg length discrepancy affects lower limbs, pelvis and trunk biomechanics of individuals with knee osteoarthritis during gait. Clin. Biomech..

[42] Levinger P (2013). Relationship between foot function and medial knee joint loading in people with medial compartment knee osteoarthritis. J. Foot Ankle Res..

[43] Gonçalves GH (2017). Ankle strength impairments associated with knee osteoarthritis. Clin. Biomech..

[44] Andersen JL, Schjerling P, Saltin B (2000). Muscle, genes and athletic performance. Scientific American.

[45] Cornwall MW, Mcpoil TG (1994). The Influence of Tibialis Anterior Muscle Activity on Rearfoot Motion during Walking. Foot Ankle Int..

[46] Mündermann A, Dyrby CO, Andriacchi TP (2005). Secondary gait changes in patients with medial compartment knee osteoarthritis: Increased load at the ankle, knee, and hip during walking. Arthritis Rheum..

[47] Ruan MZC, Patel RM, Dawson BC, Jiang M-M, Lee BHL (2013). Pain, motor and gait assessment of murine osteoarthritis in a cruciate ligament transection model. Osteoarthr. Cartil..

[48] Adães S (2014). Intra-articular injection of collagenase in the knee of rats as an alternative model to study nociception associated with osteoarthritis. Arthritis Research & Therapy.

[49] Pingel J (2017). Injection of high dose botulinum- toxin A leads to impaired skeletal muscle function and damage of the fibrilar and non-fibrilar structures. Sci. Rep.

[50] Yin Z (2017). Progressive Motor Deficit is Mediated by the Denervation of Neuromuscular Junctions and Axonal Degeneration in Transgenic Mice Expressing Mutant (P301S) Tau Protein. J. Alzheimers. Dis..

[51] Makii Y (2018). Or thopaedic Surger y Alteration of gait parameters in a mouse model of surgically induced knee osteoarthritis..

[52] Jacobs BY (2018). The Open Source GAITOR Suite for Rodent Gait Analysis. Sci. Rep.

[53] Silva JM (2018). Muscle wasting in osteoarthritis model induced by anterior cruciate ligament transection. PLoS One.

[54] Tsai H-C, Chen T-L, Chen Y-P, Chen R-M (2017). Traumatic osteoarthritis-induced persistent mechanical hyperalgesia in a rat model of anterior cruciate ligament transection plus a medial meniscectomy. J. Pain Res.

[55] Bondeson, J., Blom, A. B., Wainwright, S., Hughes, C. & Caterson, B. The Role of Synovial Macrophages and Macrophage-Produced Mediators in Driving Inflammatory and Destructive Responses in Osteoarthritis. **62**, 647–657 (2010). 10.1002/art.27290 20187160

[56] Lange-brokaar BJED (2012). Synovial in fl ammation, immune cells and their cytokines in osteoarthritis: a review. Osteoarthr. Cartil..

[57] Wood, M. J. *et al*. Macrophage proliferation distinguishes 2 subgroups of knee osteoarthritis patients Find the latest version: Macrophage proliferation distinguishes 2 subgroups of knee osteoarthritis patients. **4**, 0–12 (2019). 10.1172/jci.insight.125325 PMC6413777 30674730

[58] Stoop R (2001). Type II collagen degradation in articular cartilage fibrillation after anterior cruciate ligament transection in rats. Osteoarthr. Cartil..

[59] Dias C, Vasilceac F, Durigan J, Medeiros A, Mattiello S (2014). Analysis of local and systemic TNF-a and IL1-a expression in the acute phase of knee osteoarthritis of rats. Cytokine J..

[60] Lehrer BGM, Ornstein L (1959). A Diazo Coupling Method for the Electron Microscopic Localization of Cholinesterase. Cell.

[61] Deschenes MR (2000). Effects of resistance training on neuromuscular junction morphology. Muscle nerve.

[62] Pissulin CNA (2017). GaAs laser therapy reestablishes the morphology of the NMJ and nAChRs after injury due to bupivacaine. J. Photochem. Photobiol. B Biol..

[63] Livak KJ, Schmittgen TD (2001). Analysis of Relative Gene Expression Data Using Real-Time Quantitative PCR and the 2−ΔΔCT Method. Methods.

[64] Ramirez C (2011). Joint inflammation alters gene and protein expression and leads to atrophy in the tibialis anterior muscle in rats. Am. J. Phys. Med. Rehabil..

[65] Quadros AU (2015). Dynamic weight bearing is an efficient and predictable method for evaluation of arthritic nociception and its pathophysiological mechanisms in mice. Sci. Rep..

[66] Pinto LG (2015). Joint production of IL-22 participates in the initial phase of antigen-induced arthritis through IL-1beta production. Arthritis Res. Ther..

[67] Talbot J (2015). CCR2 expression in neutrophils plays a critical role in their migration into the joints in rheumatoid arthritis. Arthritis Rheumatol..

[68] Pastoureau PC, Hunziker EB, Pelletier J (2010). Cartilage, bone and synovial histomorphometry in animal models of osteoarthritis. Osteoarthr. Cartil..

[69] Renner AF (2013). Muscle Stretching after Immobilization Applied at Alternate Days Preserves Components of Articular. Cartilage..

[70] Gerwin N, Bendele ZAM, Glasson S, Carlson CS (2010). The OARSI histopathology initiative e recommendations for histological assessments of osteoarthritis in the rat. Osteoarthr. Cartil..

