# Supplementary material for: Knee osteoarthritis induces atrophy and neuromuscular junction remodeling in the quadriceps and tibialis anterior muscles of rats
Source: Sci Rep. 2019 Apr 24;9:6366. doi: 10.1038/s41598-019-42546-7 (PMC6482306; doi:10.1038/s41598-019-42546-7)
Supplement: Supplementary file 1 — Supplementary table 1, Supplementary table 2, Supplementary table 3, Supplementary table 4, Supplementary table 5; Supplementary Figure 1; Supplementary Figure 2 [file 41598_2019_42546_MOESM1_ESM.docx]

**Knee osteoarthritis induces atrophy and neuromuscular junction remodeling in the quadriceps and tibialis anterior muscles of rats**

Jonathan Emanuel Cunha¹, PT, MSc; Germanna Medeiros Barbosa¹, PT, MSc; Paula Aiello Tomé de Souza Castro¹, PhD; Beatriz Leite Ferreira Luiz¹, PT; Andreza Cristine Arcari Silva¹, PT; Thiago Luiz Russo¹, PT, MSc, PhD; Fernando Augusto Vasilceac², MSc, PhD; Thiago Mattar Cunha³, MSc, PhD; Fernando Queiróz Cunha³; Tania Fátima Salvini¹, PT, MsC, PhD.

**Author Affiliations:** ¹Physical Therapy Department and ²Gerontology Department, Federal University of São Carlos, São Carlos, SP, Brazil. ³ Pharmacology Department, University of São Paulo, Ribeirão Preto, SP, Brazil.

**Corresponding Author:** Tania F. Salvini, PT, MSc, PhD. Neuromuscular Plasticity Laboratory, Physical Therapy Department, Federal University of São Carlos, Rodovia Washington Luiz, Km 235, CEP: 13.565-905, São Carlos, SP, Brazil (tania@ufscar.br)

**Running title:** Neuromuscular changes in Knee OA

**Supplementary Table 1.** Loss of animals

| **Loss of animals during the study** | | |
| --- | --- | --- |
| **Reason for loss** | **n = Control group** | **n = KOA group** |
| Death during surgery | 0 | 1 |
| Death during evolution of the KOA | 2 | 1 |

**Supplementary Table 2.** Gait analysis

| **Gait test** | | | | | | |  |  |  |  |
| --- | --- | --- | --- | --- | --- | --- | --- | --- | --- | --- |
|  | **Stride Length**  **(cm)** | **Base**  **(cm)** | **Paw Length**  **(cm)** | **Paw Angle**  **(°)** | **Paw Width**  **(cm)** | **Paw Area**  **(cm²)** |  |  |  |  |
| **Control** | 14.00 ± 0.47 | 2.25 ± 0.11 | 3.56 ± 2.04 | 90.57 ± 1.98 | 2.14 ± 0.05 | 4.43 ± 0.17 |  |  |  |  |
| **KOA** | 11.25 ± 0.71** | 1.95 ± 0.08 | 3.15 ± 0.34 | 89.23 ± 0.79 | 1.97 ± 0.02* | 3.93 ± 0.20* |  |  |  |  |

Abbreviations: KOA = Knee osteoarthritis. Data are expressed as mean ± SEM. *p <0.05 and **p<0.001, KOA group compared to controls.

**Supplementary Table 3.** Mankin score

| **Modified Makin histological scores** | | | | | |
| --- | --- | --- | --- | --- | --- |
|  | **Structure** | **Cellularity** | **Safranin-O** | **Tidemark** | **Final Score** |
| **Control 1** | 1 | 0 | 0 | 0 | **1** |
| **Control 2** | 0 | 0 | 0 | 0 | **0** |
| **Control 3** | 0 | 0 | 1 | 0 | **1** |
| **Control 4** | 0 | 0 | 0 | 0 | **0** |
| **Control 5** | 0 | 0 | 1 | 0 | **1** |
| **Control 6** | 0 | 0 | 1 | 0 | **1** |
| **Mean group 0.66 ± 0.5** | | | | | |
| **KOA 1** | 0 | 1 | 1 | 0 | **2** |
| **KOA 2** | 1 | 1 | 1 | 0 | **3** |
| **KOA 3** | 0 | 1 | 1 | 0 | **2** |
| **KOA 4** | 2 | 2 | 2 | 1 | **7** |
| **KOA 5** | 1 | 1 | 1 | 0 | **3** |
| **KOA 6** | 3 | 2 | 2 | 1 | **8** |
| **Mean group * 4.16 ± 2.6** | | | | | |

Abbreviations: KOA = Knee osteoarthritis. Data are expressed as mean ± SEM. *p <0.05, KOA group compared to controls.

**Supplementary Table 4.** Modified Histological-Histochemical Mankin Grading

| **Histological-Histochemical Grading** | |
| --- | --- |
| I – Structute |  |
| a)     Normal | 0 |
| b)    Surface irregularities | 1 |
| c)    Pannus and surface irregularities | 2 |
| d)    Clefts to transitional zone | 3 |
| e)    Clefts to radial zone | 4 |
| f)    Clefts to calcified zone | 5 |
| g)    Complete disorganization | 6 |
| II – Cells |  |
| a)     Normal | 0 |
| b)    Diffuse hypercellularity | 1 |
| c)     Cloning | 2 |
| d)    Hypocellularity | 3 |
| III **-**  Safranin-O staining |  |
| a)     Normal | 0 |
| b)    Slight reduction | 1 |
| c)     Moderate reduction | 2 |
| d)    Severe reduction | 3 |
| e)     No dye noted | 4 |
| IV **-**  Tidemark |  |
| a)     Intact | 0 |
| b)    Crossed by blood vessels | 1 |

**Supplementary Table 5.** Oligonucleotide primers used for real-time PCR amplification of reverse transcribed RNA

| **Gene** | **Primer sequence** |
| --- | --- |
| HPRT | Forward: CTCATGGACTGATTATGGACAGGA  Reverse: GCAGGTCAGCAAAGAACTTATAGC |
| GAPDH | Forward: TGCACCACCAACTGCTTA  Reverse: GGATGCAGGGATGATGTTC |
| ACTB | Forward: CAGGTCATCACTATCGGCAATG  Reverse: TTTCATGGATGCCACAGGATTC |
| Pplb | Forward: TCTCGGAGCGCAATATGAAGG  Reverse: AGCAAAAGGAAGACGACGG |
| Ppla | Forward: TGGCAAATGCTGGACCAAAC  Reverse: TGCCTTCTTTCACCTTCCCAA |
| Atrogin-1 | Forward: TACTAAGGAGCGCCATGGATACT  Reverse: GTTGAATCTTCTGGATCCAGGAT |
| MuRF-1 | Forward: TGACCAAGGAAAACAGCCACCAG  Reverse: TCACTCCTTCTTCTCGTCCAGGATGG |
| MuSK | Forward: TAATGTGCAAAAGGAAGACG  Reverse: TTACAAAGGAACCAAAGGTG |
| Agrin | Forward: CTGGGACGAGGACTCAGAAG  Reverse: AGGGACACTCTGGCAGCTAA |
| α-nAChR | Forward: TCCCTTCGATGAGCAGAACT  Reverse: AGCCGTCATAGGTCCAAGTG |
| ε-nAChR | Forward: CCGAGGTCTTCTCTCCACAG  Reverse: ACCACCAAGACGTCACCTTC |
| γ-nAChR | Forward: AACGCAAGCCCCTCTTCTAT  Reverse: GAGGATCGCAACTGAGGAGA |


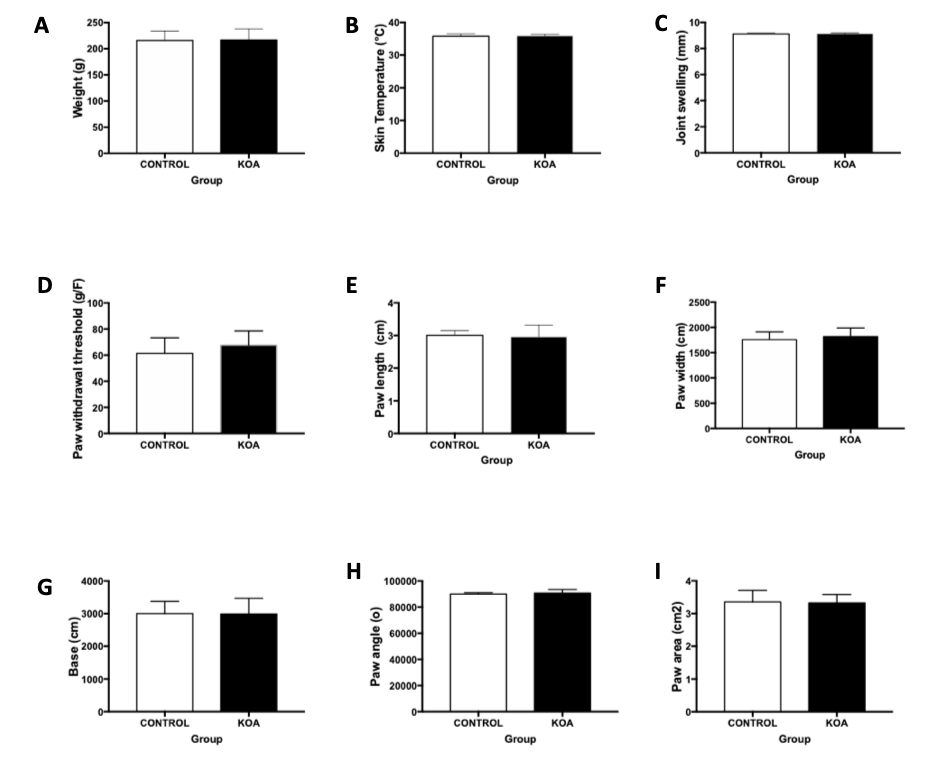


**Supplementary Figure 1.** Baseline analysis. Results from baseline (day 0) showing no intergroup difference before the ACLT. Weight (A); Thermography (B); Joint swelling (C); Paw withdrawal threshold (D); Paw length (E); Paw width (F); Paw base (G); Paw angle (H); Paw area (I). KOA = Knee osteoarthritis.

**Supplementary Figure 2**: Full-length blots. KOA = Knee osteoarthritis; MuRF-1 = Muscle Ringer finger 1; N-CAM = Neural cell adhesion molecule. The blots were performed on the same membranes after striping with β-mercaptoethanol.
